# A Simplified Sablik’s Approach to Model the Effect of Compaction Pressure on the Shape of Hysteresis Loops in Soft Magnetic Composite Cores

**DOI:** 10.3390/ma13010170

**Published:** 2020-01-01

**Authors:** Adam Jakubas, Krzysztof Chwastek

**Affiliations:** Faculty of Electrical Engineering; Częstochowa University of Technology, 42-201 Częstochowa, Poland; adam.jakubas@gmail.com

**Keywords:** soft magnetic composites, magnetic properties, Jiles–Atherton model, Sablik model

## Abstract

A novel approach to take into account the effect of compaction pressure on the shape of modeled hysteresis curves of self-developed soft magnetic composite cores is presented. The description relies on the introduction of an additional term in the so-called effective field, which is assumed proportional to the compaction pressure. The proposed model bears some resemblance to the Sablik’s extension of the Jiles–Atherton model, readily used in the studies of the magnetoelastic effect. Verification of the description is carried out using measurement data from self-developed iron-based composite cores.

## 1. Introduction

The magnetoelastic effect in ferromagnetic materials is of crucial interest to practitioners interested in exploring new possibilities for development of novel sensors and high-performance machines [1,2,3,4,5]. From the historical perspective, the magnetostriction effect, i.e., the change in shape of a ferromagnetic body under the action of external magnetic field, was one of the oldest coupled phenomena studied already by J. P. Joule in 1842 [6]. The complementary effect, i.e., the change of magnetization in ferromagnetic materials subject to applied forces, either tensile or compressive, was described by E. Villari in 1865 [7]. Since these two coupling effects are very important for the performance of electromagnetic devices, for a long time they have been the subject of considerable interest to engineers and physicists [1,3,4,5,8,9,10,11,12].

An important step towards better understanding of the magnetoelastic coupling and its effect on the shape of the hysteresis loop was made in the eighties of the last century, when D. C. Jiles and D. L. Atherton developed a simple model of ferromagnetic hysteresis [13,14]. The Jiles–Atherton (JA) model was capable of taking into account the magnetomechanical effect by the introduction of an additional term in the so-called effective field, being an indispensable part of the description. Subsequently M. Sablik and co-workers have scrutinized the possibility to consider the magnetoelastic effect in hysteresis loop modeling [15,16,17]. The introduction of magnetoelastic effect has been implemented in the JA model by consideration of an additional term in the effective field, which appears explicitly in model equations. From the engineering perspective the effective field is understood as a means to introduce the results of any phenomenon into a theoretical model. In this way an approximation of the effect is obtained. The effective field should be perceived as a cooperative interaction between numerous contributions that amplify the action of external stimulus. Generally speaking, the effective field may include the effects of eddy currents $H_{\omega }$, thermal viscosity $H_{T}$, mechanical stresses $H_{\sigma }$, demagnetization effects $H_{D}$, etc., which may be written as [18]

(1) $$H_{\mathrm{eff}}=H+H_{\omega }+H_{T}+H_{\sigma }+H_{D}.$$

In the present paper we consider the effective field as consisting of Weiss’ mean field term and the magnetoelastic term, i.e., $H_{\mathrm{eff}}=H+\alpha M+H_{\sigma }$, where the last term is attributed in the literature to M. Sablik. The Weiss’ mean field term αM takes into account mutual interactions between magnetic moments within the material [19,20,21]. According to the well-known monograph [22] (page 130), “… it is of invaluable importance in giving a simple and at the same time deep physical interpretation of the existence of spontaneous magnetization …”.

It should be stated that the Jiles–Atherton (JA) model [13], despite some deficiencies that shall be discussed in the subsequent part of the manuscript, still remains one of the most commonly used descriptions of hysteresis loops. This is most probably due to its relatively simple implementation and the possibility to take different physical phenomena into account. Description of magnetomechanical coupling is one of the most important application targets [23]. The Jiles–Atherton model including the magnetoelastic term is usually applied for the description of magnetization processes in steels [15,16,17,24,25,26,27,28,29,30]. Papers devoted to other materials of practical importance like amorphous and nanocrystalline alloys [31,32] or ferrites [33] are less common.

In one of the landmark papers Sablik et al. [15] focused on the choice of the most appropriate expression for the λ=λ(M,σ) dependence. The paper [16] attempted to correlate magnetostriction λ to such physical quantities as Burgers’ vector, Poisson’s ratio and Young’s modulus. Later Sablik provided some clues how to combine some ideas inherent in his and the Schneider–Cannell–Watts [34] descriptions of magnetomechanical hysteresis in order to explain the Villari reversal [17]. Jianwei Li and Minqiang Xu followed this line of reasoning and obtained a good agreement with experiment [24]. Lo et al. [25] studied the interrelating effects of plastic deformation and stress on magnetic properties of a series of nickel samples, which were pre-stressed to various plastic strain levels. An important conclusion from their study was that the value of model parameter k was dependent on the applied stress (this parameter is approximately equal to coercive field strength; the aforementioned conclusion was later used in modeling residual stresses in drawn wires [35] and an indirect proof of its correctness may be inferred from the analysis of real-life experimental data in Reference [36]). The paper [26] included an in-depth analysis of the relationships between strain-hardening stress and micro-structural quantities such as dislocation density and some values of JA model parameters. In a subsequent study Jianwei Li et al. [27] suggested that yet another term representing the contribution due to residual stresses in the expression for the effective field should be accounted. Jiancheng Leng et al. used the Jiles–Atherton–Sablik (JAS) model to explain variations of magnetic memory signals caused by early stages of plastic deformation [28]. Singh et al. [29] analyzed the effect of stress on hysteresis loops of non-oriented electrical steel with the JAS model. In their approach magnetostriction was modeled as a product of two functions, λ=f(M)g(σ). The first function was a polynomial, the second one was a hyperbolic tangent with offset. The authors were able to describe the magnetoelastic effects in the examined steel with a reasonable accuracy. Quite recently Hergli et al. [30] suggested that the JA model parameter a might be related to plastic deformation. However, in their work they availed of the classical JA model without the Sablik’s term in the expression for the effective field.

In the present paper we focus on the possibility of applying the JAS model to self-developed soft magnetic (SMC) cores. The JA model was used previously for the SMC materials by Benabou et al. [37], Zidarič and Miljavec [38] and by Ślusarek et al. [39]. The paper [37] compared the capabilities of classical JA model to the Preisach approach. The authors of [38] suggested that the reversibility parameter *c* should be made dependent on the excitation amplitude. The paper [39] analyzed the dependence of model parameters on processing temperature for commercial Somaloy 500 samples. The modified JA model [40] was used in modeling. However, none of the papers [37,38,39] used the extended expression for the effective field with the magnetoelastic term. The present paper was written to fill the gap advancing a more complete description. 

In this work we attempted to model hysteresis loops of self-developed SMC cores subject to different compaction pressures, whose effect on the loop shapes was assumed to be described with the Sablik’s term in the effective field.

## 2. The JAS Model Equations

The set of equations considered in this work were as follows:

(2) $$\frac{dM}{dH_{\mathrm{eff}}}=\frac{\delta_{M}\left(M_{\mathrm{an}}-M\right)}{k\delta },$$

(3) $$H_{\mathrm{eff}}=H+\alpha M+\frac{3}{2}\frac{\sigma }{\mu_{0}}\left(\frac{d\lambda }{dM}\right)≅H+\alpha M+K\sigma M,$$

(4) $$M_{\mathrm{an}}=M_{s}\left[\coth\left(\frac{H_{\mathrm{eff}}}{a}\right)-\frac{a}{H_{\mathrm{eff}}}\right].$$

Equation (2) defines the relationship between magnetization and the effective field. According to model developers, their intention was to introduce an expression similar to that used for description of dry friction phenomenon. The next expression defines the effective field, which in our paper included an additional term assumed to be proportional to the applied (and residual) stress. Equation (3) defines the so-called anhysteretic magnetization curve, which was one of the building blocks for the model. Theoretically, the anhysteretic curve should describe an idealized material devoid of pinning sites, which, according to Jiles and Atherton, are responsible for the occurrence of hysteresis, since they hamper the domain wall motion within the sample. However, at this point we would like to point out, that there are serious interpretational problems for the afore-mentioned relationship in Equation (4), discussed in detail in Reference [41].

The model parameters $\alpha ,a,k,M_{s},K.$ δ=±1 were used in order to distinguish the upper and lower loop branches whereas $\delta_{M}=0.5\left[1+\mathrm{sign}\left(\left(M_{\mathrm{an}}-M\right)\cdot dH/dt\right)\right]$ was introduced in order to suppress the negative susceptibilities obtained after sudden field reversals [41,42,43]. The assumed form of model equations was similar to the one considered in the original paper [13]. 

In this paper we made a number of simplifying assumptions. We did not take the reversible magnetization component into account in model equations (introduced in the subsequently published most-cited paper on the JA model, i.e., Reference [44]), in order to avoid the subtle intricate conceptual problems with the JA model, these have been addressed thoroughly in other papers [41,42,43,45,46]. We assumed a linear dependence of magnetostriction on magnetization, since some problems were reported for more complicated relationships [47]. Finally, we did not make the assumption that the value of the k parameter might be varied in dependence on the applied stress, as suggested in Reference [25]. During modeling we kept the values of all parameters fixed, contrary to the approach presented in Reference [48]. The only mechanism that made the model sensitive to stress was the simplified (linear) functional dependence in the last term of the expression in Equation (3) that defines the so-called effective field. In order to justify our approach we referred to a very interesting remark made on the role of mathematical models by P. W. Anderson in his Nobel prize lecture from 1977, cited in Reference [49]: “Very often such, a simplified model throws more light on the real workings of nature than any number of “ab initio” calculations of individual situations, which even where correct often contain so much detail as to conceal rather than reveal reality. It can be a disadvantage rather than an advantage to be able to compute or to measure too accurately, since often what one measures or computes is irrelevant in terms of mechanism. After all, the perfect computation simply reproduces Nature, does not explain her.” We believe that the simplified JAS model should be able to describe at least qualitatively the change of shape of hysteresis loops of self-developed SMC cores. 

After transformations described in detail in References [50,51] the expression for dM/dB is derived:

(5) $$\frac{dM}{dB}=\frac{\delta_{M}\left(M_{\mathrm{an}}-M\right)}{\mu_{0}\left[k\delta +\left(1-\alpha^{*}\right)\left(M_{\mathrm{an}}-M\right)\right]}$$

where the stress dependent parameter is $\alpha^{*}=\alpha +K\sigma$. It is explicitly dependent on the stress σ applied to the sample during its processing. As the result of material compaction, the iron grains are squeezed together which results in an improved packing ratio for the considered soft magnetic composite (SMC) cores. Smaller air and insulator gaps between the iron grains imply that the effect of demagnetization fields becomes less significant [52,53]. We considered that the residual stresses induced in the material after compaction were in the first approximation proportional to the compaction pressure applied to the sample.

Equation (5) may be integrated to yield hysteresis curves taking into account the effect of compaction pressure on the shape of the loops. The values of field strength in the corresponding time instants are computed from the constitutive relationship, $H(t)=B(t)/\mu_{0}-M(t).$ The assumption of constant value for coefficient K makes the considered model equivalent to the Schneider–Cannell–Watts description [34]. The necessity to transform model equations in order to derive explicitly the expression for dM/dB stems from the fact that in magnetic measurements the controlled variable is magnetic flux density, whose waveform should be kept sine [54].

## 3. Measurements, Modeling

**Figure 1:**
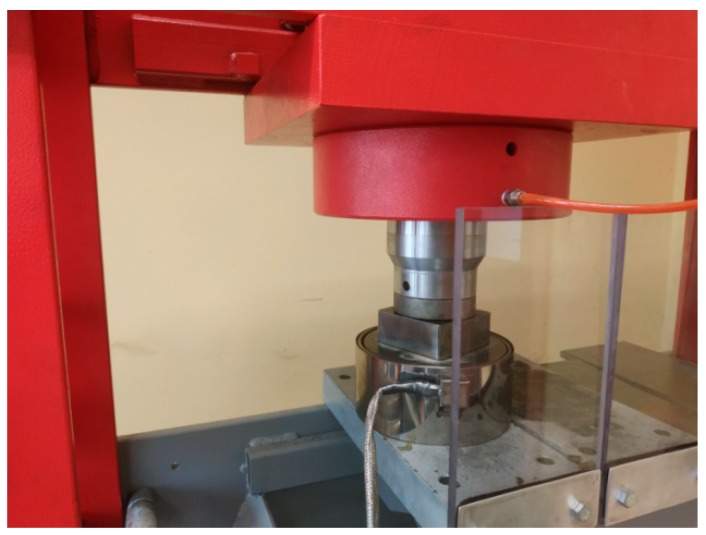
Mechanical press used for sample preparation.

**Figure 2:**
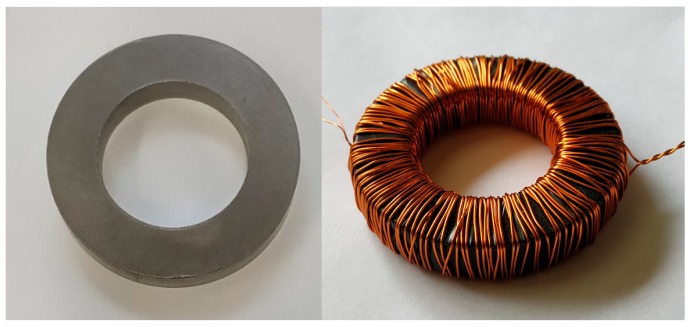
Exemplary cores and measuring transformer prepared by compacting Fe powder and polyvinyl chloride (PVC).

A mixture of iron powder (99% pure Fe, granulation 100–150 μm) and polyvinyl chloride PVC-S (granulation 15–100 μm) was prepared in order to develop some SMC cores for tests. Comparable grain size of both constituents allowed us to obtain a highly homogenous mixture. Polyvinyl chloride used as the matrix material has good mechanical properties. Moreover, it is resistant to many solvents and it is a good electrical insulator. From the obtained mixtures cylinder-shaped samples were formed using a hydraulic press with a mold and a heating band for controlling the heat treatment conditions. Figure 1 depicts the press device used during sample preparation. The powder mixture was backfilled into the mold and subsequently pressed for five minutes at a temperature of 175 °C. This step was aimed at making the mixture plasticized. The difference between the developed samples relied on the application of different force/pressure during their forming. Five samples were developed, for them the range of the applied stamp forces/pressures was from 10 T (78 MPa) up to 65 T (507 MPa). Figure 2 presents some of the developed cores. The core dimensions were: the outer radius—25 mm, the inner radius—15 mm, whereas the height was equal to 10 mm. Some samples were found to be brittle, therefore we focused in subsequent studies on those formed at pressures >310 MPa. Next the measurements of magnetic properties were carried out. The primary (100 coils) and the secondary (30 coils) windings were wound on the examined SMC cores.

**Figure 3:**
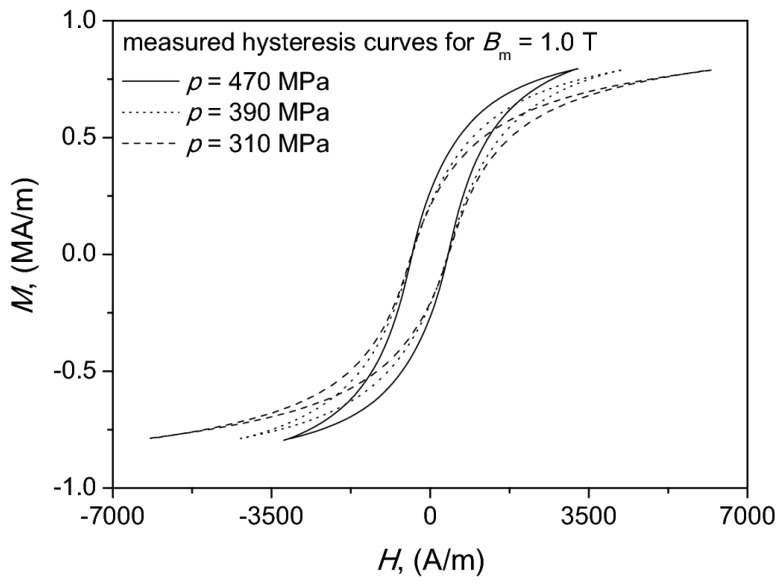
Measured hysteresis curves for chosen values of compaction pressure.

The weight percentage ratio Fe powder vs. PVC was kept constant at 99.5/0.05. We noticed that for compaction pressure equal to 470 MPa the obtained maximum induction was approximately 1.3 T, which is a value comparable to the one for some permalloys or amorphous alloys. For lower compaction pressures *B*_max_ values were lower. We have chosen as the representative value *B*_m_ = 1.0 T in order to depict the shapes of some measured hysteresis curves in Figure 3. 

**Figure 4:**
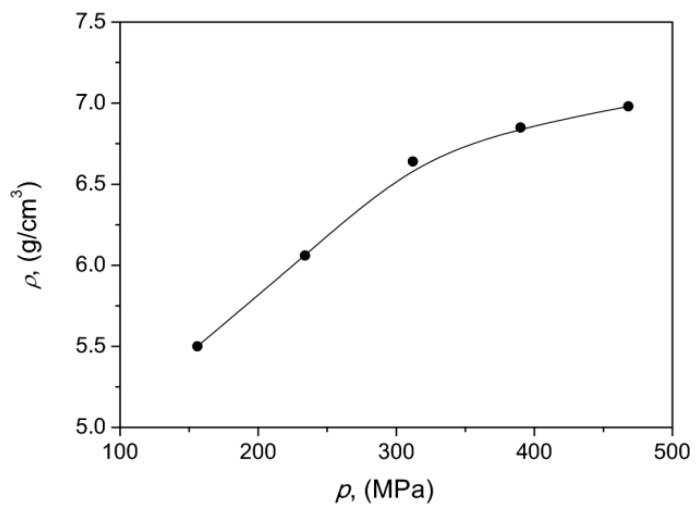
Experimental dependence of material density versus compaction pressure.

In the next step we determined experimentally how the density of the developed SMC cores depended on the compaction pressure. The appropriate dependence is depicted in Figure 4. It can be stated that as the compaction pressure increased, the material density also increased, which implied better magnetic properties due to a higher packing ratio. The results are consistent with those obtained in Reference [55] for commercial Somaloy. A qualitatively similar dependence (exhibiting saturation after a certain threshold value) was presented in Reference [56]. In the subsequent ρ=ρ(p) analysis we focused on the range of compaction pressures p≥300 MPa, which implied improved magnetic properties.

The density measurements were carried out using the buoyancy method. In order to determine density for a solid specimen from the definition, ρ=m/V, it is necessary to know the sample mass and volume. For solids with irregular shapes, the volume may be determined using the so-called hydrostatic scales method, whose working principle is based on the Archimedes law. For measurements one uses fluids with known density (e.g., distilled water with $\rho_{w}=$1000 kg/m³ at 4 °C and with $\rho_{w}=$997.07 kg/m³ at 25 °C). The measurement relied on weighing the samples twice, at first in the air (sample weight denoted then as G_1_), and in the second case in the fluid (sample weight denoted as G_2_). The difference between both values was equal to the buoyancy force $W=\rho_{w}Vg$. The sought value of density was determined from the relationship $\rho =\rho_{w}G_{1}/\left(G_{1}-G_{2}\right)$.

**Figure 5:**
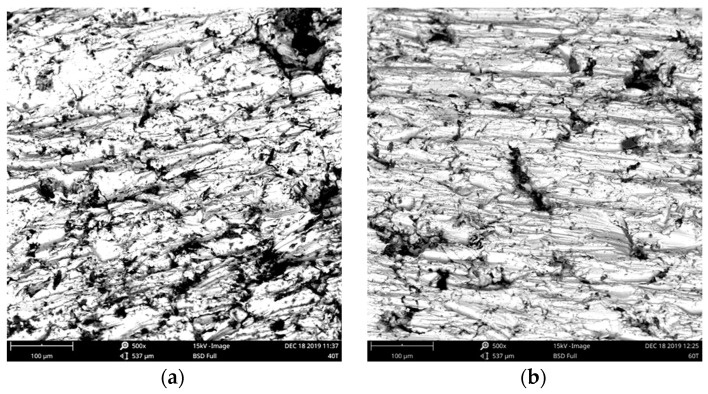
Cross-section microstructure photographs (**a**) for compaction pressure 310 MPa, (**b**) for compaction pressure 470 MPa.

**Figure 6:**
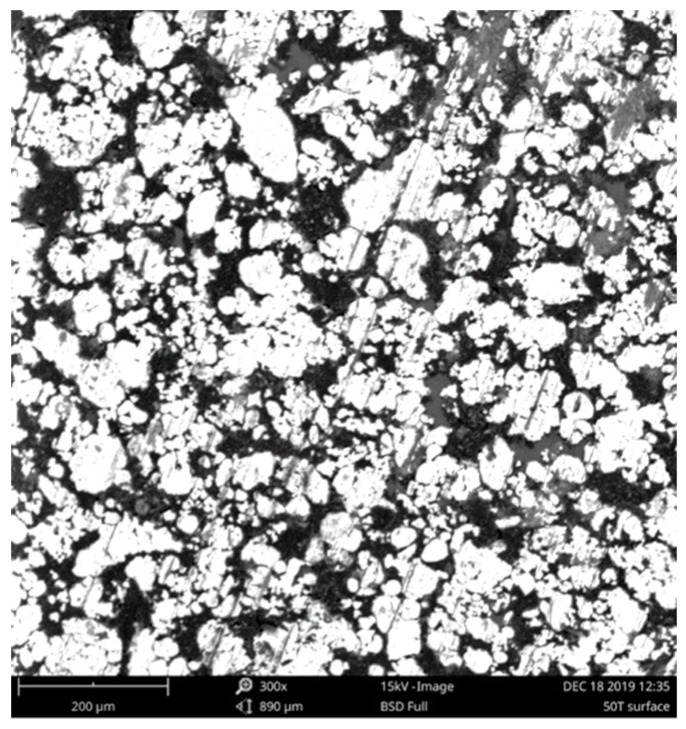
Surface microstructure photograph for compaction pressure 390 MPa.

Figure 5 depicts the images of cross-section microstructures for the examined samples from the scanning electron microscope Phenom ProX (ThermoFisher Scientific, Waltham, MA, USA): subfigure a) for compaction pressure 310 MPa (pressure load 40 tons), b) for compaction pressure 470 MPa (pressure load 60 tons). The white areas are iron grains, the gray ones are polymer matrix, whereas the darkest spots are air gaps. From the figure it is visible that the increase of compaction pressure resulted in decreased volume of air gaps in the SMC material. Moreover, it can be noticed from the figure that iron grains were subject to flattening and formed layers under compaction pressure. For comparison, in Figure 6 the microstructure of the surface for the sample subject to compaction pressure 390 MPa (pressure load 50 tons). 

**Figure 7:**
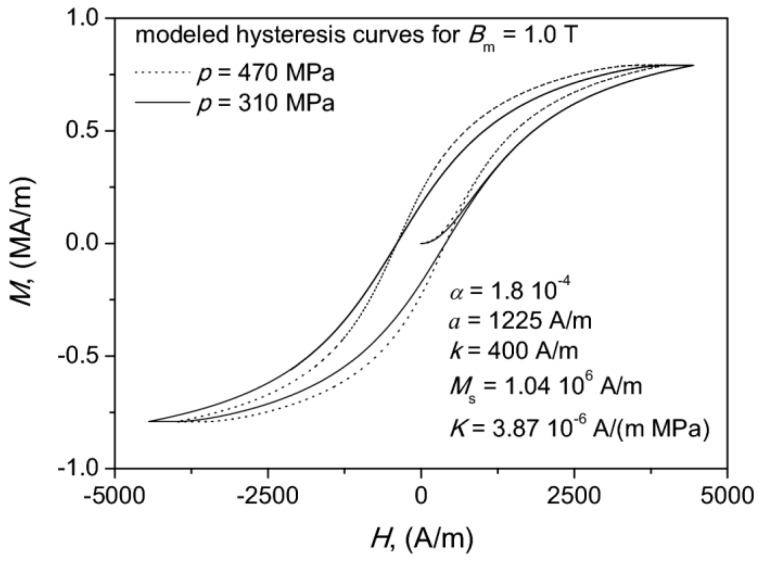
Modeled hysteresis curves for *B_m_* = 1.0 T and the estimated set of Jiles–Atherton–Sablik (JAS) model parameters.

The JAS model parameters were estimated using the robust DIRECT algorithm [57]. Their values as well as some chosen modeled hysteresis curves are shown in Figure 7. We noticed that generally the model underestimated the remanence values. The error in determination of coercive field strength did not exceed 2.4%. For remanence point it was around 16.5%. This value might seem to be too high at first glance, but we would like to point out that the scale at the ordinate axis is several orders of magnitude bigger than at the abscissa axis. The averaged deviation of the modeled and the measured data points for the descending (upper) branch of the considered hysteresis loops for 310 MPa and 470 MPa did not exceed 10% and 7.7%, respectively. The deviation for a single data point was computed from the relationship $\delta_{\%}=100\cdot \left|\left(M_{\mathrm{model}}-M_{\mathrm{meas}}\right)/M_{\mathrm{meas}}\right|$.

In order to get some insight on the usefulness of the results, we also computed the areas covered by the measured and the modeled hysteresis curves in order to estimate the modeling error for power loss density from the definition of the hysteresis loop area. For compacting pressure p=310 MPa the model overestimated the value of power loss density by 33%, whereas for p=470 MPa—by 21.4%.

It can be stated that a qualitative change of shape of modeled curve was possible to be obtained by updating the value of the effective mean-field parameter.

Using the same values of model parameters modeling was carried for a lower induction amplitude, $B_{m}$ = 0.6 T. As already stated, in the present paper we assumed that the values of all model parameters were kept fixed. However, it should be remarked that in the past a lot of research on the JA model was devoted to examination of model accuracy for lower excitation levels, to mention as representative examples References [40,58,59,60]. In the above-mentioned references the necessity to update some values of model parameters upon the excitation amplitude is raised and different functional relationships are proposed as a method to improve the model accuracy. In particular the model parameter k, interpreted as related to the average energy required to break the pinning sites (and taking the values comparable to coercive field strength), is found to vary upon excitation level.

**Figure 8:**
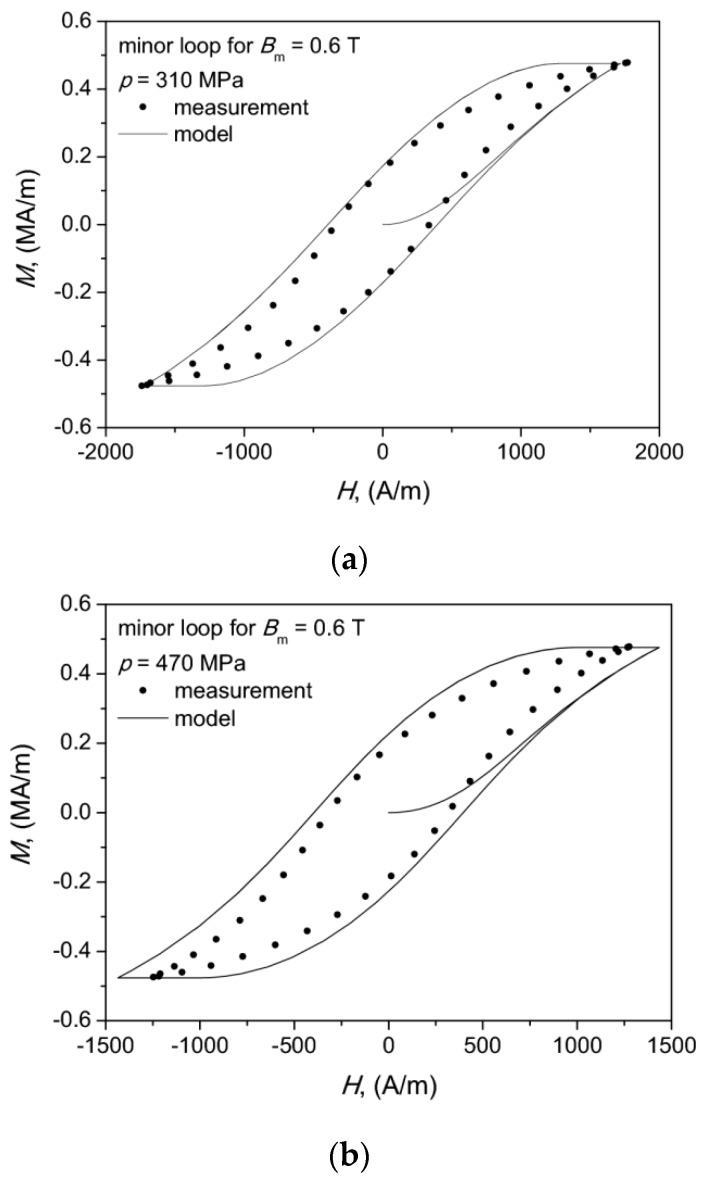
Modeled hysteresis curves for *B_m_* = 0.6 T: case (**a**)—for 310 MPa, case (**b**)—for 470 MPa. Dots denote measurement points.

The modeling results for our simplified approach with fixed values of model parameters for minor loops are shown in Figure 8. For the considered SMC material a reasonable modeling accuracy was obtained without any parameter value update, which can be qualitatively assessed from the aforementioned figure. The modeling error for the characteristic points in the M(H) plane did not exceed 25%.

An additional conclusion that can be drawn from the analysis of the obtained results is that the modeled values of coercive field strength for the considered samples were roughly the same, whereas the modeled values of remanence magnetization exhibited a clear dependence on the compaction pressure. Similar qualitative observations were made previously by one of us in a study focused on the possibility to use the Jiles–Atherton–Sablik model for determination of residual stress in drawn wires [35].

## 4. Conclusions

In the paper we have applied the Jiles–Atherton–Sablik model to describe hysteresis curves of self-developed SMC cores compacted at different pressures. The effect of varying compaction pressure was accounted as an additional term in the so-called effective field. We have kept the values of model parameters fixed. In order to obtain a stress-dependent model extension we have introduced a functional dependence on stress in one term defining the effective field. The results might be of interest to the designers of magnetic cores.
